# A Case Report of Coronary Artery Stenosis Induced by Ablation for Premature Ventricular Complexes

**DOI:** 10.7759/cureus.71764

**Published:** 2024-10-18

**Authors:** McKenna Schaar, Alaina Werling, Simon Tran, Jyoti Mohanty

**Affiliations:** 1 Internal Medicine, Nova Southeastern University Dr. Kiran C. Patel College of Osteopathic Medicine, Fort Lauderdale, USA; 2 Interventional Cardiology, Palm Beach Gardens Medical Center, Palm Beach Gardens, USA

**Keywords:** cabg, catheter ablation, left main coronary artery stenosis, pci, pvc, sinus of valsalva

## Abstract

Many patients with premature ventricular complexes (PVCs) do not experience symptoms. However, for those with frequent or symptomatic PVCs, medical interventions are available. If medications fail, radiofrequency catheter ablation may be performed. Despite careful attention to avoiding the coronary arteries during ablation, injuries may still occur. Here, we present the case of a 66-year-old Caucasian female with frequent PVCs causing worsening palpitations, chest pain, fatigue, and lightheadedness, who underwent elective PVC ablation and suffered catheter-induced left main coronary artery stenosis, necessitating subsequent coronary artery bypass grafting (CABG). This case highlights a rare complication of cardiac ablation in the setting of PVCs and the rationale for clinical decisions made throughout the patient's hospital course.

## Introduction

A premature ventricular contraction (PVC) refers to an additional, irregular heartbeat originating in the ventricles. PVCs are prevalent in the general population and are estimated to occur in up to 75% of adults undergoing prolonged cardiac monitoring [1]. In most cases, PVCs occur spontaneously [2]. Factors contributing to increased PVC frequency include advanced age, male gender, hypertension, and a history of heart disease [2-3]. The exact pathophysiology of PVCs is still unknown. Three mechanisms that are recognized as possible pathways include triggered activity, automaticity, and reentry [3]. Many patients with PVCs are asymptomatic. Those with symptoms commonly complain of having the sensation of a 'skipped' heartbeat. This likely occurs due to the pause between a PVC and the next regular heartbeat. Additional presenting symptoms may include palpitations, chest discomfort, dyspnea, lightheadedness, and, rarely, syncope [2]. Initial evaluation includes a 12-lead electrocardiogram. However, due to the infrequent nature of PVCs in some patients, a 24- or 48-hour Holter monitor or long-term event monitor may be required for accurate diagnosis. Further diagnostic steps may include blood work, echocardiography, and stress testing. Asymptomatic patients typically do not require treatment for PVCs. In patients experiencing frequent or symptomatic PVCs with unidentifiable etiology, medical interventions are available. These include antiarrhythmics such as amiodarone or flecainide, beta-blockers, and calcium channel blockers. Radiofrequency catheter ablation may be performed in the case of medication failure. Catheter ablation is generally a safe procedure, with a complication rate ranging from 3.1% to 5.2%. A key factor influencing complication risk is the ablation site, with higher rates observed in procedures involving the left ventricle and epicardial regions. Potential complications include coronary artery occlusion and AV block [4-5]. Here, we present a 66-year-old Caucasian female who underwent elective PVC ablation and suffered from catheter-induced left main coronary artery stenosis. We also discuss the clinical decisions made throughout her hospital course.

## Case presentation

A 66-year-old Caucasian female with a medical history of hypothyroidism, prior supraventricular tachycardia (SVT) ablation, and frequent premature ventricular contractions presented for elective catheter PVC ablation. She reported experiencing 'skipped beats' for several years, with worsening palpitations, chest pain, fatigue, and lightheadedness. A beta-blocker trial was unsuccessful, leading to symptomatic bradycardia. EKG showed frequent PVCs. An echocardiogram (ECHO) showed mild cardiomyopathy with an ejection fraction (EF) of 45%, mild mitral regurgitation, and trivial tricuspid regurgitation. A 24-hour Holter monitor showed a 40% PVC burden, unifocal. Given the patient's presentation, work-up, and failure of medical management, she was promptly scheduled for PVC ablation.

**Figure 1:**
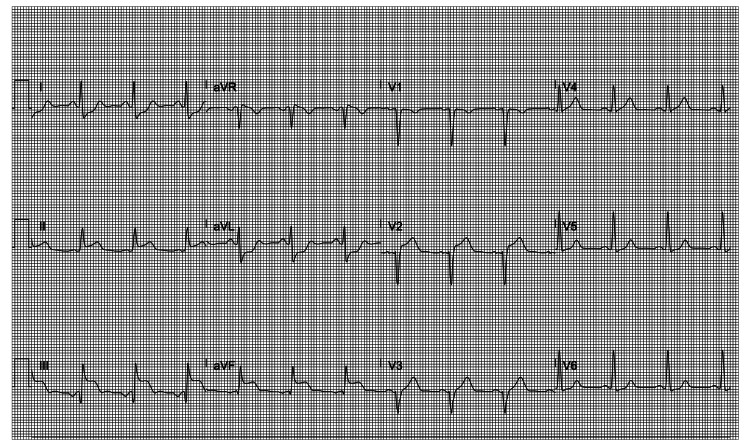
ST elevation in leads I and aVL with concurrent ST depression in the inferior leads during isoproterenol infusion.

**Figure 2:**
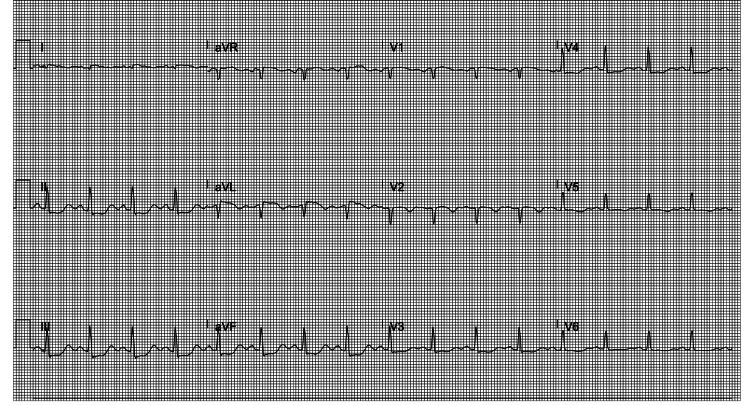
Worsening ST changes post-procedure.

Under the care of an electrophysiologist, the patient was prepared for PVC ablation. Moderate sedation was administered by the anesthesia team, and the procedure started thereafter. Venous and arterial access was obtained under direct ultrasound guidance. An intracardiac echocardiography (ICE) catheter was inserted and advanced to the mid-right atrium. A comprehensive survey was done with the creation of right ventricular outflow tract (RVOT) and left ventricular outflow tract (LVOT) contours. The ostium of the left main coronary artery was marked. Subsequently, a SmartTouch SF ablation catheter was used for PVC mapping in the left ventricular (LV) summit by activation and pace mapping, with the earliest point of activation being 10-15 ms pre-QRS in the LVOT. Ablation was performed here at 40-50W with no suppression of PVCs. An ablation catheter was then retrogradely placed into the LV and was used to map the LVOT. Ablation was performed at 50W in the left and right sinuses of Valsalva and left-right commissure. Special attention was paid to avoid coronary arteries, with impedance monitored using ultrasound visualization. Following ablation, PVCs were drastically reduced. High-dose isoproterenol was infused with rare PVCs during washout. During infusion, the patient was noted to have some ST elevation in I and aVL with concurrent ST depression in the inferior leads (Figure 1). Serial EKGs demonstrated improvement. However, upon waking, the patient reported chest tightness. Another EKG was performed, revealing worsening ST changes despite administration of nitroglycerin (Figure 2). Interventional cardiology was consulted and agreed that coronary angiography was indicated.

Emergent left and right coronary angiography were performed using standard catheters. This revealed severe left main disease with over 90% stenosis at its ostium with TIMI 2 flow (Video 1). All branches of the left coronary remained patent, as well as the right main and its branches (Video 2). A repeat angiography of the ostial left main was conducted using a 5 Fr catheter, and 200 mcg of intracoronary nitroglycerin was given to alleviate any spasm. No clots were identified. There was severe damping of pressure at the ostial level once multiple views were obtained, suggesting severe ostial stenosis. An LV angiogram was performed with a 6 Fr pigtail catheter, revealing moderately reduced LV contractility, with an EF of 35% and wall motion abnormality. The aortic root appeared normal, with no evident aortic regurgitation. Given the reduced EF, wall motion abnormality, slow flow, and hypotension, an intra-aortic balloon pump (IABP) was placed to improve coronary flow and the patient was initiated on heparin. Following this, the patient reported improved chest pain, achieved hemodynamic stability, and EKGs normalized. Cardiac surgery was consulted, and after reviewing the films, concurred that the patient was a suitable candidate for coronary artery bypass graft (CABG).

**Video 1:** Left coronary angiography revealing severe left main disease. Source: Dr. Schaar M.

**Video 2:** Right coronary angiography. Source: Dr. Schaar M.

Two bypass grafts were performed. A reverse saphenous vein to obtuse marginal branch anastomosis was performed, followed by a left internal mammary artery to left anterior descending artery (LAD) anastomosis. A transesophageal echocardiogram (TEE) demonstrated stable right ventricular function with left ventricular ejection fraction (LVEF) improved to 40-45%. The patient otherwise tolerated the procedure well and was transferred to the ICU in stable condition.

## Discussion

Premature ventricular complexes are a frequently encountered cardiac occurrence, with up to a 75% rate of incidence in the general population [1]. Many individuals may experience PVCs without awareness. However, a subset of the population contends with a heightened PVC burden, manifesting symptoms such as palpitations, chest discomfort, dyspnea, lightheadedness, or syncope [2]. Patients displaying these symptoms undergo a comprehensive evaluation.

Primary intervention for individuals with a significant PVC burden and associated symptoms with no identifiable cause involves initiating a trial of medication. In cases where medication proves ineffective, patients may then be referred for cardiac ablation [3]. Cardiac ablation uses radiofrequency energy to destroy small areas of cardiac tissue thought to be responsible for originating the PVCs. The selection of the ablation site relies on electrical activity mapping to determine the location of ectopic activity that will most effectively eliminate PVCs.

The majority of PVC origins are typically localized in the RVOT [4]. However, in our patient, mapping and ablation were initially performed at the left ventricular summit, unfortunately yielding no suppression of PVCs. Subsequently, ablation was performed at the left and right sinuses of Valsalva and left-right commissure, leading to near-complete resolution of PVCs. Each of the aortic sinuses of Valsalva is situated in close proximity to atrial and ventricular myocardium, rendering ablation of these sites effective for challenging or difficult-to-access areas in endocardial ablation [6]. Both initial and long-term success rates of sinus of Valsalva ablation are high. Site-specific complications may include coronary artery occlusion or AV block [4]. Despite careful attention to avoid the coronary arteries, injuries may still occur, as evidenced in our patient who suffered from catheter-induced left main coronary artery stenosis.

Following the discovery of thermal injury during coronary angiography, the decision was made to proceed with coronary artery bypass grafting (CABG). Given the ongoing debate between CABG and percutaneous coronary intervention (PCI) for left main coronary artery (LMCA) disease, several critical factors were considered prior to making this decision. This included the type and location of the injury, as well as the overall stability of the patient.

The American Heart Association (AHA) currently recommends CABG for individuals requiring revascularization due to significant left main involvement associated with high-complexity coronary artery disease [7]. However, for isolated ostial disease, PCI has comparable long-term outcomes to CABG [8]. All of this is to be considered within the context of coronary artery disease caused by atherosclerosis.

As in our case, an endothelial burn is largely unpredictable in nature and the evolution of the patient’s lesion could not be anticipated [9]. Despite her low Syntax score, it is possible that PCI may have proved inadequate for treatment or caused further complications. Moreover, our patient experienced resolution of symptoms and hemodynamic stability after the placement of an intra-aortic balloon pump (IABP). This, combined with her unremarkable past medical history and low STS score, made her a suitable candidate for CABG with a low likelihood of needing revascularization [10]. Had the patient been unable to regain hemodynamic stability, emergent stent placement would have been performed.

## Conclusions

This case highlights a rare complication of cardiac ablation in the setting of PVCs, resulting in left coronary artery ostium stenosis. Currently, insufficient research exists to determine the optimal treatment approach between CABG and PCI for such cases. By sharing this case, we aim to encourage further research to assist physicians in making informed clinical decisions for optimal patient outcomes.

## References

[1] Ahn MS (2013). Current concepts of premature ventricular contractions. J Lifestyle Med.

[2] Farzam K, Richards JR (2023). Premature ventricular contraction. http://www.ncbi.nlm.nih.gov/books/NBK532991/.

[3] Marcus GM (2020). Evaluation and management of premature ventricular complexes. Circulation.

[4] Tada H (2012). Catheter ablation of tachyarrhythmias from the aortic sinuses of Valsalva--when and how?. Circ J.

[5] Wang JS, Shen YG, Yin RP (2018). The safety of catheter ablation for premature ventricular contractions in patients without structural heart disease. BMC Cardiovasc Disord.

[6] Rillig A, Meyerfeldt U, Birkemeyer R, Jung W (2009). Ablation within the sinus of Valsalva for treatment of supraventricular and ventricular tachycardias: what is known so far?. Europace.

[7] Virani S, Newby L, Arnold S (2023). 2023 AHA/ACC/ACCP/ASPC/NLA/PCNA Guideline for the management of patients with chronic coronary disease: a report of the American Heart Association/American College of Cardiology Joint Committee on Clinical Practice Guidelines. Circulation.

[8] Park S, Park SJ, Park DW (2022). Percutaneous coronary intervention for left main coronary artery disease: present status and future perspectives. JACC Asia.

[9] Klaudel J, Trenkner W, Glaza M, Miekus P (2019). Analysis of reported cases of left main coronary artery injury during catheter ablation: In search of a pattern. J Cardiovasc Electrophysiol.

[10] Fosbøl EL, Zhao Y, Shahian DM, Grover FL, Edwards FH, Peterson ED (2013). Repeat coronary revascularization after coronary artery bypass surgery in older adults: the Society of Thoracic Surgeons' national experience, 1991-2007. Circulation.

